# Multilocus microsatellite analysis of '*Candidatus *Liberibacter asiaticus' associated with citrus Huanglongbing worldwide

**DOI:** 10.1186/1471-2180-12-39

**Published:** 2012-03-20

**Authors:** Md-Sajedul Islam, Jonathan M Glynn, Yang Bai, Yong-Ping Duan, Helvecio D Coletta-Filho, Gopal Kuruba, Edwin L Civerolo, Hong Lin

**Affiliations:** 1 USDA-ARS San Joaquin Valley Agricultural Research Science Center, Parlier, CA 93648, USA; 2 Guangxi Citrus Research Institute, Gulin, Guangxi 530004, China; 3 USDA-ARS Horticultural Research Laboratory, Fort Pierce, FL 34945, USA; 4 Instituto Agronômico de Campinas, Cordeirópolis, CEP 13490-970 São Paulo, Brazil; 5 Citrus Research Station, Andhra Pradesh Horticultural University, Tirupati 517502, Andhra Pradesh, India

## Abstract

**Background:**

Huanglongbing (HLB) is one of the most destructive citrus diseases in the world. The disease is associated with the presence of a fastidious, phloem-limited α- proteobacterium, '*Candidatus *Liberibacter asiaticus', '*Ca*. Liberibacter africanus' or '*Ca*. Liberibacter americanus'. HLB-associated Liberibacters have spread to North America and South America in recent years. While the causal agents of HLB have been putatively identified, information regarding the worldwide population structure and epidemiological relationships for '*Ca*. L. asiaticus' is limited. The availability of the '*Ca*. L. asiaticus' genome sequence has facilitated development of molecular markers from this bacterium. The objectives of this study were to develop microsatellite markers and conduct genetic analyses of '*Ca*. L. asiaticus' from a worldwide collection. Two hundred eighty seven isolates from USA (Florida), Brazil, China, India, Cambodia, Vietnam, Taiwan, Thailand, and Japan were analyzed.

**Results:**

A panel of seven polymorphic microsatellite markers was developed for '*Ca*. L. asiaticus'. Microsatellite analyses across the samples showed that the genetic diversity of '*Ca*. L. asiaticus' is higher in Asia than Americas. UPGMA and STRUCTURE analyses identified three major genetic groups worldwide. Isolates from India were genetically distinct. East-southeast Asian and Brazilian isolates were generally included in the same group; a few members of this group were found in Florida, but the majority of the isolates from Florida were clustered separately. eBURST analysis predicted three founder haplotypes, which may have given rise to three groups worldwide.

**Conclusions:**

Our results identified three major genetic groups of '*Ca*. L. asiaticus' worldwide. Isolates from Brazil showed similar genetic makeup with east-southeast Asian dominant group, suggesting the possibility of a common origin. However, most of the isolates recovered from Florida were clustered in a separate group. While the sources of the dominant '*Ca*. L. asiaticus' in Florida were not clearly understood, the less-pervasive groups may have been introduced directly from Asia or via Brazil. Notably, the recent outbreak of HLB in Florida probably occurred through multiple introductions. Microsatellite markers developed in this study provide adequate discriminatory power for the identification and differentiation of closely-related isolates, as well as for genetic studies of '*Ca*. L. asiaticus'.

## Background

Huanglongbing (HLB) is one of the most devastating diseases of citrus, which is characterized by the development of yellow shoots and stunted growth of infected trees combined with a decline in quantity and quality of fruit production [1]. HLB-affected fruit are abnormally-pigmented, developmentally flawed, and have a bitter taste- making them unusable for juice production or as table fruit [2,3]. Typically, trees with HLB succumb to the effects of infection and die within a few years after showing the first signs of the disease [4].

HLB is associated with three '*Candidatus *Liberibacter' species worldwide: *'Ca*. L. asiaticus', '*Ca*. L. africanus' and '*Ca*. L. americanus'; the nomenclature is based on the presumptive origin of each bacterium in Asia, Africa and South America, respectively [1]. HLB has been known in Asian countries since the 1870s [1,5,6] and found to be associated with the presence of a fastidious α-proteobacterium named '*Candidatus *Liberibacter asiaticus'. In the western hemisphere, it was reported in São Paulo, Brazil in 2004 and in Florida, USA in 2005- two of the largest citrus growing regions in the world [1]. Although '*Ca*. L. americanus' initially constituted a major proportion of the total bacterial population in Brazil, this ratio has changed since 2004, and *'Ca*. L. asiaticus' is now the most prevalent citrus-destroying species [4]. Both '*Ca*. L. americanus' and *'Ca*. L. asiaticus' are transmitted by a psyllid vector, *Diaphorina citri *(also known as the Asian citrus psyllid, or ACP) in Asia, North America, and South America [7,8]. The HLB-associated Liberibacters can also be transmitted by grafting propagative material from infected plants onto nursery stock. The continued economic losses associated with HLB are a serious threat to the U.S. citrus industry [9]. HLB affects all citrus cultivars [10] and to date there are no known HLB-resistant citrus cultivars.

The genetic structure within a given pathogen population can be a valuable resource for determining the source or origin of the pathogen and risk management of the disease. The availability of a genome sequence for '*Ca*. L. asiaticus' has provided information on the metabolic features of this bacterium and key insights into HLB pathogenesis [11]. In addition, the genome sequence has facilitated the development of DNA markers for genetic analysis; these molecular genetic markers are critical for understanding the genetic diversity of global populations and the epidemiology of HLB. DNA markers have been used for characterization of microbial populations associated with plant diseases, including RAPDs, SNPs, MLST and SSRs (microsatellites) [12-15]. Molecular genetic markers not only aid in the general characterization of a given population, but can help identify the source of an introduced pathogen [16].

Among the three HLB-associated Liberibacter species, '*Ca*. L. asiaticus' is the most widespread and is responsible for increasing economic losses of citrus industries. Much attention has been drawn by researchers in the last few years to the importance of understanding the epidemiology and ecology of the disease associated with '*Ca*. L. asiaticus'. '*Ca*. L. asiaticus' isolates were characterized in some previous studies; most of these studies focused on the Asian continent and utilized conserved genes as genetic markers. For example, southeast Asian isolates were characterized by sequencing the 16S rDNA and 16S/23S regions, *omp*, the *rpl *gene cluster, and the bacteriophage-type DNA polymerase [17]. The 16S rDNA was employed for understanding genetic diversity of '*Ca*. L. asiaticus' in India [18] and a prophage gene was used to reveal variations in China [19]. However, genetic variation within conserved genes has limited discriminatory power to differentiate closely-related isolates within populations.

Microsatellite DNA markers associated with hypervariable sequence regions can provide sufficient resolution for differentiating closely-related isolates and for tracking genotypes of interest; additionally, these markers may help identify the source of invasive strains. Recently, similar types of markers have been used for differentiating '*Ca*. L. asiaticus' in Japan [20]. Chen et al. [21] studied populations from Guangdong province in China and Florida in the United States. However, the single variable locus used in that study provided limited characterization of '*Ca*. L. asiaticus' genetic diversity.

Here, we report a panel of seven polymorphic microsatellite markers for conducting genetic analyses of '*Ca*. L. asiaticus' isolates from Asia (India, China, Cambodia, Vietnam, Thailand, Taiwan, and Japan), North America (Florida, USA) and South America (São Paulo, Brazil). The microsatellite profile for each isolate was compared with all members of the sample set to make predictions on the possible origin and dissemination of HLB in Florida.

## Results

### PCR amplification and characteristic of microsatellite loci

A total of 287 '*Ca*. L. asiaticus' isolates obtained from HLB-affected plants across Asia and the Americas (Additional file 1 and Table 2) were successfully amplified by all seven microsatellite primer pairs. The number of alleles and haploid genetic diversity per locus ranged from 2 to 30, and 0.204 to 0.881, respectively (Table 1). In the clone-corrected data set, genotypic linkage disequilibrium was not detected by pairwise comparison of loci across the overall isolates (*P *> 0.01).

**Table 1:** Characteristics of seven microsatellite markers developed from '*Candidatus *Liberibacter asiaticus'

SSR Markers	Primer sequences (5'-----3')	Repeats	Location in genome	ORF	*T*_a _(°C)	Size range (bp)	No. of alleles	*H*
LasSSR-A-f LasSSR-A-r	**FAM**-CGCCTACAGGAATTTCGTTACG TCTCATCTTGTTGCTTCGTTTATCC	(TATTCTG)_8_	255477-255753	adenosine deaminases	50°C	241-434	30	0.881
LasSSR-B-f LasSSR-B-r	**VIC**-ATCGCCTATAAATCCCTTTACTGATATGTTTCC TGGTAACGGAAGTGATAATAACTACAGCAATAAG	(TTTAA)_6_	669257-669458	hypothetical protein	60°C	196-206	3	0.216
LasSSR-C-f LasSSR-C-r	**VIC**-CGATTGTTGATGAATTACC GAATAGAAGAACCCTAAGC	(CAGT)_8_	666722-666947	phosphohydrolases	50°C	208-254	15	0.613
LasSSR-D-f LasSSR-D-r	**NED**-CGGTGTCGGTATCGGTATCATTC CGAAGAAGAGACGGAGGTTAAGC	(TTC)_5_	377678-377850	hypothetical protein	55°C	158-174	3	0.391
LasSSR-E-f LasSSR-E-r	**NED**-GATCAGTAGTCTATCACCAC TACTGGAAACAAATGGAATAC	(CTTGTGT)_5_	354424-354613	transcriptional regulator	50°C	173-290	17	0.587
LasSSR-F-f LasSSR-F-r	**FAM**-TCGTCTTATCGTATATCACTCC TTCACTATTAAAGGATCAAGGC	(TTTACATC)_3_	520542-520307	repair ATPase	52°C	227-235	2	0.204
LasSSR-G-f LasSSR-G-r	**FAM**-CGGGAGAAATTAAAGATGATGG CGCTGTTAATACATACTTACGC	(TTGTTGGA)_2_	998251-998403	hypothetical protein	53°C	139-152	2	0.204

*T*_a_, annealing temperature of the primer pairs; H, Haploid genetic diversity

Each forward primer was labeled with FAM, NED, VIC fluorescent dyes at 5', respectively

**Table 2:** Descriptive statistics and genetic diversity of '*Candidatus *Liberibacter asiaticus' isolates across seven microsatellite loci in the samples obtained from nine different countries from Asia, North (Florida, USA) and South Americas (São Paulo, Brazil)

Country	Location ID	Location Information	Total number of individuals	Number of individuals in clone corrected data	Alleles per locus	Haploid genetic diversity
**Brazil**	BRA	São Paulo	**22**	**14**	**2.7**	**0.313**
**USA**	FL-A	Charlotte County, Florida	5	4	1.6	0.161
	FL-B	Collier County, Florida	46	11	2.1	0.234
	FL-C	DeSoto County, Florida	30	5	1.7	0.194
	FL-D	Hardee County, Florida	8	5	1.7	0.160
	FL-E	Hendry County, Florida	13	5	1.6	0.171
	FL-F	Highlands County, Florida	19	6	1.7	0.119
	FL-G	Indian River, County, Florida	23	7	1.9	0.175
	FL-H	Martin County, Florida	10	7	1.9	0.175
	FL-I	Okechobee County, Florida	4	2	1.3	0.143
	FL-J	Polk County, Florida	6	4	2.0	0.304
	FL-K	St. Lucie County, Florida	6	4	1.4	0.179
	FL-L	Pasco County, Florida	2	2	1.1	0.071
	FL-M	Manatee County, Florida	2	2	1.3	0.143
	FL-N	Hillsborough County, Florida	2	2	1.3	0.071
	FL-O	Lake County, Florida	1	1	1.0	0.000
	**USA-Florida-overall**		**177**	**67**	**3.6**	**0.247**
**CHINA**	CHN-A	Baise, Guangxi Province	3	2	1.1	0.071
	CHN-B	Guilin, Guangxi Province	3	3	1.4	0.190
	CHN-C	Hezhou, Guangxi Province	3	2	1.3	0.143
	CHN-D	Nanning, Guangxi province	14	14	3.0	0.276
	CHN-E	Fuzhou, Fujian Province	7	5	2.1	0.320
	CHN-F	Tangshan, Yunnan Province	3	2	1.3	0.143
	CHN-G	Gangzhoou, Jiangxi Province	1	1	1.0	0.000
	CHN-H	Guangzhou, Guangdong Province	1	1	1.0	0.000
	CHN-I	Hunan Province	1	1	1.0	0.000
	**China-overall**		**36**	**31**	**5.7**	**0.342**
**CAMBODIA**	CAM-A	Pursat Province	7	6	2.4	0.341
	CAM-B	Battambang Province	4	4	1.9	0.304
	**Cambodia-overall**		**11**	**10**	**3.1**	**0.423**
**VIETNAM**	VIET	Hung Yen Province, Hoa Binh Province, Hanoi	**3**	**3**	**1.9**	**0.317**
**THAILAND**	THAI	Unknown	1	1	1.0	0.000
**TAIWAN**	TIW	Unknown	1	1	1.0	0.000
**JAPAN**	JPN	Unknown	1	1	1.0	0.000
**INDIA**	IND-A	Anantapur District, Andhra Pradesh	7	7	2.4	0.297
	IND-B	Chittoor District, Andhra Pradesh	6	6	2.0	0.254
	IND-C	Kadapa District, Andhra Pradesh	4	4	1.9	0.250
	IND-D	Mahaboobnagar District, Andhra Pradesh	3	3	1.4	0.159
	IND-E	Nalgonda District, Andhra Pradesh	4	4	1.7	0.196
	IND-F	Prakasam District, Andhra Pradesh	4	3	2.4	0.540
	IND-G	Tirupati District, Andhra Pradesh	5	5	2.0	0.274
	IND-H	Kurnool District, Andhra Pradesh	1	1	1.0	0.000
	IND-I	Ludhiana District, Punjab	1	1	1.0	0.000
	**India-overall**		**35**	**34**	**5.4**	**0.360**

Allele per locus: average number of alleles per locus

Clone corrected data (removed repeated genotypes within a population)

### Genotype and genetic diversity

A total of 117 genotypes (haplotypes) were identified (Additional file 1). Haplotypes identified within the sample population were restricted to the boundaries of their country of origin. The genetic diversity observed in different countries and locations are summarized in Table 2. Isolates from China possessed the largest number of alleles (5.7 alleles per locus), followed by India (5.4 alleles per locus). Overall haploid genetic diversities were the highest in Asian countries, followed by Brazil. The haploid genetic diversity of the Florida (USA) isolates was lowest among all the geographic groupings (Table 2).

### Genetic structure

**Figure 1:**
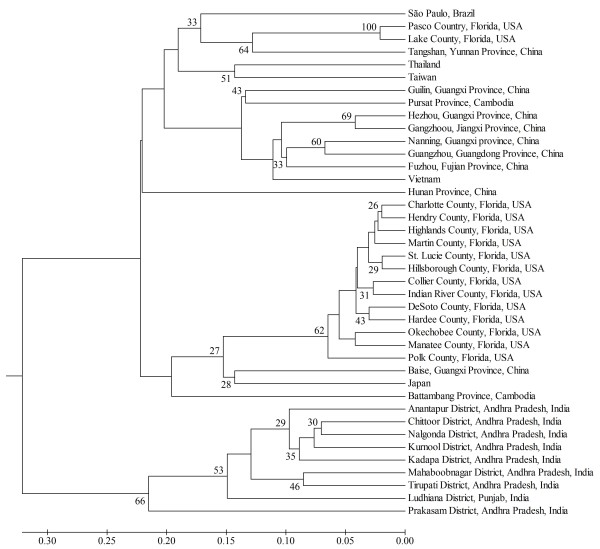
**UPGMA dendrogram showing the genetic relationships of '*Candidatus *Liberibacter asiaticus' isolates from different locations within an individual country as well as from different countries (from Asia and Americas)**. Clone-corrected data were used for constructing the dendrogram based on DA distance [22]. Only bootstrap values > 25% are shown.

A UPGMA clustering analysis identified three major groups of '*Ca*. L. asiaticus' (Figure 1). Isolates from India were clustered in a distinct group (group 3). Most of the isolates from China and other Asian countries, and Brazil were generally grouped in group 1. While some isolates from Florida occurred in group 1, most isolates from Florida were clustered in group 2 (Figure 1).

**Figure 2:**
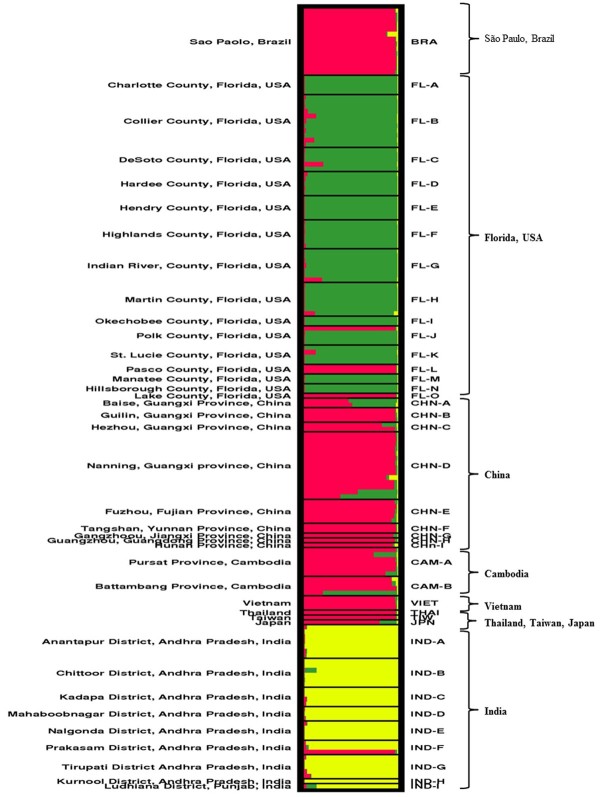
**Individual assignments of '*Candidatus *Liberibacter asiaticus' isolates obtained from nine different countries from Asia and Americas by STRUCTURE analysis**. There were three clusters (K). Black lines within the squares distinguish geographic locations.

The STRUCTURE analysis based on Bayesian modeling further assessed the genetic structure of '*Ca*. L. asiaticus'. This approach utilizes statistical methods to determine the relationships among the isolates without prior population information. In the analysis three different clusters (K) were identified based on the *ad hoc *statistic ΔK [23] (Figure 2). The membership of each individual isolate obtained from STRUCTURE analysis, can be estimated as (*q*), the ancestry coefficient, which varies on a scale between 0-1.0, with 1.0 indicating full membership in a population. Individuals can be assigned to multiple clusters (with values of *q *summing to 1.0) indicating they are admixed. Individual samples with *q *≥ 0.90 (ancestry coefficient) were considered as having single lineage and individuals with *q *< 0.90 were considered as admixed lineages as followed by Williams et al. [24]. The result of STRUCTURE analysis is consistent with UPGMA in which isolates from India were grouped in a distinct cluster (Figure 2 in yellow). Brazilian and most east-southeast Asian isolates were clustered as a single lineage (*q *≥ 0.90) (Figure 2, red). Some isolates taken from central Florida (Polk, Pasco, and Lake Counties) shared the same lineage with east-southeast Asian and Brazilian isolates (Figure 2, red). Most Florida isolates, however, grouped in a different cluster (Figure 2, green). Some admixed isolates (*q <*0.90) were found in Florida as well as in Baise and Nanning of Guangxi province in China, and in Cambodia.

**Figure 3:**
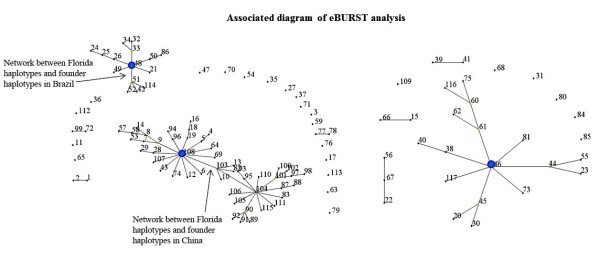
**Network diagram (based on nearly identical haplotypes that differed by two loci) from eBURST analysis**. Solid blue circles in the diagram indicate three predicted founder haplotypes: China (Haplotye-108), Brazil (Haplotype-48) and India (Haplotype-46). A primary network was observed between haplotype-103 and 107 (Florida), and predicted founder haplotypes in China, and between haplotype-51 (Florida) with predicted founder haplotypes in Brazil, suggesting two separate introductions of '*Ca*. L. asiaticus' into Florida.

eBURST analysis with user-defined criteria (based on the analysis of haplotypes that shared identical genotypes for at least 5 of the 7 loci) predicted three founder haplotypes: haplotype-108 (Nanning, Guangxi province, China), haplotype-48 (São Paulo, Brazil) and haplotype-46 (Tirupati District, Andhra Pradesh, India) (Additiontal file 1 and Figure 3). The diagram generated by eBURST showed a primary network between haplotype-103 and 107 (Collier County, Florida) and predicted founder haplotype in China. A primary network was also identified with haplotype-51 (Pasco County, Florida) and the second predicted founder haplotype in Brazil. Haplotype-46 from Tirupati District, Andhra Pradesh, India) was predicted to be the third founder and hypothesized to be the founder haplotype of '*Ca*. L. asiaticus' in India.

## Discussion

Characterization of worldwide and regional *'Ca*. L. asiaticus' populations from HLB-affected plants can facilitate identification of introduction patterns and predict the possible relationships of HLB-associated Liberibacters found in different citrus growing regions. Multilocus microsatellite marker analysis can provide sufficient resolution for differentiating closely-related isolates and can be useful for tracking genotypes of interest; additionally, these markers may help identify the source of invasive strains.

In this study, seven microsatellite markers successfully genotyped '*Ca*. L. asiaticus' from global populations. Sequence analysis indicated that three of the microsatellites appear to overlap with microsatellites recently developed by Katoh et al. [20]. Various microsatellite length variations were found in '*Ca*. L. asiaticus' from worldwide collections, with some loci having as many as 30 alleles.

Historical evidence reviewed by da Graça [25] suggested that HLB was observed in Guangdong province, China in the late 19th century [26], and later spread to other parts of the country. It is assumed that HLB may have been introduced into China from India along sea trade routes [27]. The first record of HLB-like symptoms, referred to as 'dieback', was reported from India in the 18th century [28]; this was later suggested to be HLB [29]. As '*Ca*. L. asiaticus' has been in Asian countries over a century, the genetic diversity in Asian populations was expected to be high, due to a longer period of mutation accumulation, population differentiation and natural selection. As hypothesized, a higher degree of genetic diversity for '*Ca*. L. asiaticus' was observed in both China and India within the present study (Table 2). In contrast, the lower level of allelic and haploid genetic diversity of '*Ca*. L. asiaticus' in Florida and Brazil populations are consistent with the hypothesis that '*Ca*. L. asiaticus' populations in these regions have been derived from recent introductions [30].

Human movement of infected plant materials is probably the main cause of long distance dissemination of both '*Ca*. L. asiaticus'-positive psyllids and HLB-affected plant material. The distributions of haplotypes observed in '*Ca*. L. asiaticus' in this study did not detect any identical haplotypes from different continents or even from different countries within the same continent (Additional file 1). This result does not exclude the possibility of contemporary migration of '*Ca*. L. asiaticus' among different countries through the movement of infected plant materials or by the migration of vector psyllids as rapid mutation and selection could lead to deviation of populations from their original sources. The vector, *D. citri*, has been in Brazil for over 60 years without any sign of HLB until its discovery on 2004 [4,25]. *D. citri *was discovered in Florida in Palm Beach, Broward and Martin counties in 1998 and has spread throughout the state since that time [7]. However, it is not clear when '*Ca*. L. asiaticus' was introduced into Brazil and Florida. The rapid spread of HLB in Brazil and Florida after the first reports in 2004 and 2005, respectively, suggests that HLB was most likely introduced around that time, rather than evolving from an indigenous source.

As suggested by the historical evidence and review of the early literature related to HLB, the most ancient population of '*Ca*. L. asiaticus' perhaps originated in India. From the 20^th ^century onward, HLB spread through much of the citrus-growing regions of south and southeast Asia [2], the Arabian peninsula [31], East Timor and Papua New Guinea [32], and the western hemisphere (Brazil and the United States) [1]. It is difficult to precisely know when the disease entered each country and from where it was introduced. Frequent shipment of plant materials and unlawfully importation of plants has increased the risk of disseminating exotic plant pathogens around the world. The exact pathways responsible for introducing HLB and the Asian citrus psyllid into the United States and Brazil have not yet been determined.

The genetic relationships of the isolates in this study, as determined from the UPGMA based on Nei's genetic distance [22] and individual based clustering analysis by the STRUCTURE analyses, consistently identified three major genetic groups of '*Ca*. L. asiaticus', with isolates from India included in a distinct genetic group (Figure 2 and Figure 3). The similar genetic makeup amongst most isolates from east-southeast Asia and South America (São Paulo, Brazil) support the hypothesis of the introduction of '*Ca*. L. asiaticus' into South America from East Asia or Southeast Asia. While most isolates from Florida were clustered within a separate group, both UPGMA and STRUCTURE analyses showed that some isolates from central Florida overlapped with east-southeast Asian and Brazilian groups. The presence of two genetic groups in Florida suggests at least two introduction events are associated with the recent outbreak of HLB in Florida.

Based on the history of HLB, it could be predicted that populations of '*Ca*. L. asiaticus' in Florida were most likely established following the introduction of HLB-affected plant materials or '*Ca*. L. asiaticus'-carrying psyllid from Asia or other countries through human-mediated transport. The analyses in this study do not support the hypothesis of introduction of HLB into the Americas through biological materials sourced from India. Only a single isolate from India (Prakasam District, Andhra Pradesh) overlapped with the east-southeast Asian and Brazilian group (Figure 2, red). STRUCTURE analysis revealed that less-dominant clusters (Figure 2, red) in central Florida (Polk, Pasco, and Lake Counties) were observed in the same lineage (*q *≥ 0.90) with east-southeast Asian and Brazilian clusters suggesting that the origin of members of this cluster in Florida might be derived from Asia or via Brazil. Moreover, some admixed (*q *< 0. 90) isolates between Florida and east-southeast Asia also support the hypothesis of introducing '*Ca*. L. asiaticus' into Florida from Asia.

**Figure 4:**
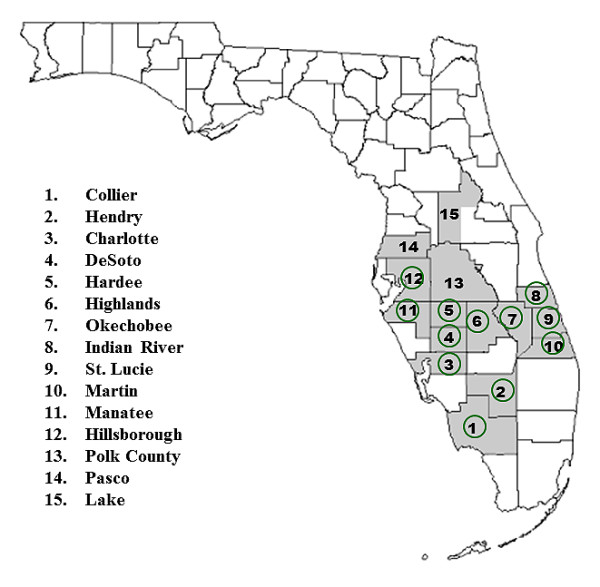
**Sample distribution of '*Candidatus *Liberibacter asiaticus' from 15 citrus-growing counties (gray highlighted) in Florida, USA**. Green circles indicate the counties where only the dominant '*Ca*. L. asiaticus' group were observed based on STRUCTURE analysis (green in Figure 2). Some isolates from Polk County (13), Pasco County (14) and Lake County (15) were included with the genetic group 2 (less dominant group) (see Figure 2).

eBURST analysis provided further insights into the origin of '*Ca*. L. asiaticus'. Founder haplotypes were identified from China, Brazil, and India. Based on their position within the eBURST network, these founders are predicted to have given rise to the three global genetic groups, consistent with prevailing theories of the geographic origins of HLB [1,2,4,7]. While one founder type was predicted in Brazil, the similar genetic makeup of Brazilian and east-southeast Asian isolates suggest that this founder could have been introduced into Brazil from any of these Asian countries. Consistent with the STRUCTURE analysis, the eBURST diagram also predicted the introduction of '*Ca*. L. asiaticus' into Florida citrus groves through at least two separate introduction events. While a primary network was detected between a founder haplotype from China and two unique haplotypes in Florida, clear differentiation was observed between most isolates from China and Florida by Bayesian clustering and UPGMA analyses. Differences between the dominant groups found in Florida and China were also reported in a recent study using a single VNTR locus [21]. It is uncertain whether the dominant group of Florida isolates were introduced *en masse *or if a small population of nearly-identical '*Ca*. L. asiaticus' haplotypes from China were introduced, evolved quickly, and established a large population. The recent discovery and rapid spread of HLB in Florida, along with wide distribution of dominant '*Ca*. L. asiaticus' group observed in the present study suggests that isolates of this group have been directly introduced from an unknown location. Another recent study also indicated that some isolates of '*Ca*. L. asiaticus' from Florida may have been introduced through two different events, and sources were unknown [21]. The analyses of microsatellites in the present study, however, suggest that the introduction of the less-dominant cluster was likely from a single source either Asia or Brazil. The low occurrence of less dominant group in some central counties in Florida suggests that the members of this group were perhaps introduced more recently (Figure 4). However, it is certainly plausible that these two haplotypes were introduced into Florida at nearly the same time. Isolates from one of the sources may have spread quickly due to selective advantage under a favorable set of biological or environmental conditions.

Our analysis showed that a dominant group of '*Ca*. L. asiaticus' genotypes are widely distributed in south-central Florida (Figure 4). HLB is highly invasive and spatial spread of the disease is rapid compared to other arboreal pathosystems [4]. Increased diversity of host, pathogen, vector, and environmental conditions likely influence the rates of HLB distribution. Moreover, the rates of HLB increase are directly related to increase and spread of the psyllid vector population: in June 1998, the Asian citrus psyllid was first detected in Palm Beach County; within two years of this discovery the disease occurred to 31 counties in Florida [33]. The vector is now present in nearly all citrus-growing areas of Florida [34]. In Florida, HLB was first discovered in Miami-Dade County in August 2005, seven years after detection of the vector in the same region [30]. By mid-October, the disease was found in many residential properties stretching northwards more than 250 km from Miami-Dade County to St. Lucie County and several commercial citrus groves were also affected in Palm Beach and Hendry Counties [1]. However, no epidemiological survey has clearly demonstrated HLB or '*Ca*. L. asiaticus'-carrying psyllids being introduced into the southern part of Florida and then spreading northward through the continuous movement of psyllid vectors. Since 2005, HLB has spread to most citrus-producing counties in Florida [34,35]. The rapid and widespread distribution of this disease among citrus growing counties in Florida is most likely due to the result of the multiple secondary introductions of HLB-associated '*Ca*. L. asiaticus'. Based on the present analyses, it appears that there were at least two '*Ca*. L. asiaticus' introduction events in Florida. Moreover, the rapid distribution of HLB within Florida after 2005 is concomitant with the discovery of a dominant genetic cluster within south-central Florida. Taken together, this suggests that dominant '*Ca*. L. asiaticus' haplotypes, possibly from different countries may have established a population within Florida through multiple introduction events.

## Conclusions

The seven microsatellites developed in this study are useful for detection, isolate differentiation, and genetic analysis of '*Ca*. L. asiaticus'. Our results showed that current '*Ca*. L. asiaticus' populations in HLB-affected citrus in Asia and the Americas are comprised of three distinct genetic groups: (1) Indian, (2) predominantly east-southeast Asian and South American (Brazil) and (3) predominantly North American (Florida, USA). While regional differences were observed from the distribution of dominant clusters, the similar genetic makeup of east-southeast Asian and Brazilian isolates lead us to hypothesize that '*Ca*. L. asiaticus' populations in Brazilian groves were most likely introduced from east or southeast Asia. The precise sources of the dominant genetic group of '*Ca*. L. asiaticus' retrieved from Florida are not clearly resolved from the present analysis. However, less-pervasive groups may have been introduced directly from Asia or via Brazil. While the results here provide some insights into the origins and genetic structure of HLB-associated '*Ca*. L. asiaticus', it should be noted that broader population analyses using a larger array of molecular markers will help resolved the questions on the origin and dissemination of HLB-associated '*Ca*. L. asiaticus.

## Methods

### Sample collection/DNA extraction

DNA from HLB-affected samples from Asia (India, China, Cambodia, Vietnam, Thailand, Taiwan, and Japan), North America (Florida, USA) and South America (State of São Paulo, Brazil) were extracted from the respective sources and sent as microbially-sterile and non-infectious samples. HLB-associated Liberibacter-free DNA samples were used as negative controls. Basically, leaf samples were collected from citrus trees with blotchy mottle and blotchy mottle-like symptoms. Leaves were washed under running tap water and blotted dry with paper towels. The midribs were then excised from the leaf blade. Total genomic DNA was extracted from 4-5 midribs per sample. Samples were ground in liquid nitrogen and DNA was extracted using the CTAB method. Precipitated DNA was dissolved in 100 μl of TE buffer. The quality of DNA samples was checked by electrophoresis in 1.2% agarose gels. DNA samples were diluted 30 times with water for PCR.

### Microsatellite marker development

To identify putative microsatellite regions in the '*Ca*. L. asiaticus' genome, we used the program 'Tandem Repeats Finder' [36]. Following the identification of these regions, primers were designed (Eurofins-Operon) that flanked the prospective repeat sequence to generate a product of 150-400 base pairs. Over 100 primer sets were tested using multiple DNA samples obtained from HLB-affected plants from India, China, Brazil and Florida. We postulated that polymorphisms, if present, should be observed within this pilot sample due to their geographic separation. Following amplification of regions containing putative microsatellite using the test primers, the products of each reaction were then run on 5% of polyacrylamide gels. Silver staining was then used to visualize polymorphic alleles. This screening procedure identified seven loci with amplified sequence length variability. To facilitate high-throughput genotyping analysis, each of seven forward primers was labelled with a fluorescent dye (Table 1). Amplified products were analyzed by an ABI 3130 *xl *Genetic Analyser (Applied Biosystems, Foster City, CA).

### PCR based genotyping

PCR was performed in 20 μl containing 2 μl of 10× reaction buffer, 1.5 mM MgCl_2_, 0.2 mM dNTPs, 0.25 U AmpliTaq Gold (Applied Biosystems, Foster City, CA), 2.5 pmole of each of SSR primer pairs and 2 μl of diluted DNA sample. PCR was conducted in the following conditions: 94°C for 4 minutes; 40 cycles consisting of 94°C for 45 seconds, annealing temperature (Table 1) for 45 seconds, and 72°C for 45 seconds; then a final extension at 72°C for 7 minutes. The successes of amplifications were checked running 5 μl of amplified products in agarose gel electrophoresis using 2.5% agarose-TBE gels. For multiplex analysis, 2 μl of each amplified product labeled with different fluorescent dye was pooled. From 8 μl of pooled product, 2.5 μl was mixed with 0.25 μl of GeneScan-500 Liz molecular size standard (Applied Biosystems Cat #4322682A) and 7.25 μl of Hi-Di Formamide (Applied Biosystems Cat. #4311320). The mixture of products was then loaded onto a Genetic Analyzer (Applied Biosystems, Foster City, CA) equipped with the 36 cm 16-capillary array filled with POP-7 polymer (Applied Biosystems, Foster City, CA). Data acquisition and fragment size determinations were carried out by GeneMapper v4.0 software (Applied Biosystems, Foster City, CA).

### Genotypes and genetic diversity analysis

Genotypes were identified based on combination of allelic data from multiloucs microsatellite loci. A clone-corrected (removing repeated genotypes within a population) data set was built and used for the analysis of genetic diversity, linkage disequilibrium and genetic structure. GenAlEx Version 6.3 [37] was used to calculate the average number of alleles (Na) and haploid genetic diversity (H) at each locus as well as across all loci for each of the populations.

### Linkage disequilibrium analysis

A global test (Fisher's method) implemented in GENEPOP web version 4.0.10 [38] was used to test for the genotyping linkage disequilibrium (LD) between all pair of loci across all samples in this study.

### Genetic structure analysis

To determine the genetic relationships of '*Ca*. L. asiaticus'isolates, a UPGMA dendrogram was constructed based on Nei's genetic distance [22]. The trees were calculated using POPULATION software package Version 1.2.31 (Olivier Langella, CNRS UPR9034, France found at web: http://bioinformatics.org/~tryphon/populations/) and graphically displayed with MEGA4 software [39]. Confidence in specific clusters of the resulting topology was estimated by bootstrap analysis with 1,000 replicates.

The program STRUCTURE 2.3.1 [40] was also used for a clustering algorithm based on a Bayesian model to assign individual isolate of '*Ca*. L. asiaticus' to a specified number of clusters. This algorithm assumes a model in which there are K clusters (where K may be unknown), each of which is characterized by a set of allele frequencies at each locus. No linkage disequilibrium was detected between all pairs of loci across all samples with the clonal corrected data set. Therefore, the program STRUCTURE 2.3.1 [40] was rationally used to estimate the number of clusters (K) within '*Ca*. L. asiaticus' where 10 independent runs of K = 1-10 were performed without any prior information as to the origin (location) of individual samples. For each run, a burn-in period of 25,000 iterations was used followed by a run length of 50,000 Markov chain Monte Carlo iterations, and a model with correlated allele frequencies and admixture among populations. The model was run with 10 independent simulations for each K. The number of clusters that best represents the observed data was determined by maximizing the estimated ln likelihood of the data for different values of K, and the ΔK index which is based on the rate of change in the ln likelihood of the data between successive K(1-10) [23]. The optimal probabilities for all individuals were estimated from 10 replicate runs at K = 3 with permutation analysis using CLUMPP version 1.1.2 [41], and the output of genetic clustering was visualized using software DISTRUCT version 1.1 [42].

To provide further insight into the relationships among '*Ca*. L. asiaticus', the eBURST v3 program http://eburst.mlst.net/ was employed to identify putative founder types. For this analysis, user- defined group definition was set to include those haplotypes that shared identical genotypes for at least 5 of the 7 loci. The minimum single-locus variant count for subgroup definition was set to 3.

## Authors' contributions

HL, MSI, JMG, YPD, HDC, GK and ELC coordinated the study, collected samples and provided preliminary data. HL, JMG, and YB carried out genotyping of HLB samples. MSI, JMG and HL analyzed results and wrote the paper. All authors read and approved the final manuscript.

## Supplementary Material

Additional file 1 **Sample and haplotype information for all isolates used in this study**. Click here for file

## References

[B1] Bové JM Huanglongbing: A destructive, newly-emerging, century-old disease of citrus J Plant Pathol 2006 88 1 7 37

[B2] da Graça JV Citrus greening disease Annu Rev Phytopathol 1991 29 109 136 10.1146/annurev.py.29.090191.000545

[B3] Baldwin E Plotto A Manthey J McCollum G Bai J Irey M Cameron R Luzio G Effect of Liberibacter infection (huanglongbing disease) of citrus on orange fruit physiology and fruit/fruit juice quality: chemical and physical Analyses J Agric Food Chem 2009 58 2 1247 1262 20030384 10.1021/jf9031958

[B4] Gottwald TR Current epidemiological understanding of citrus huanglongbing Annu Rev Phytopathol 2010 48 119 139 10.1146/annurev-phyto-073009-114418 20415578

[B5] Lin KH Yellow shoot of citrus in Chinese Acta Phytopathol Sin 1956 2 1 12

[B6] Beattie GAC Holford P Mabberley DJ Haigh AM Broadbent P On the origins of citrus, huanglongbing, *Diaphorina citri* and *Trioza erytreae* Orlando, Florida, USA: International Conference of Huanglongbing Florida 2008 25 57

[B7] Halbert SE Manjunath KL Asian citrus psyllids (Sternorrhyncha: *Psyllida*) and greening disease of citrus: a literature review and assessment of risk in Florida Fla Entomol 2004 87 330 353 10.1653/0015-4040(2004)087[0330:ACPSPA]2.0.CO;2

[B8] Manjunath KL Halbert SE Ramadugu C Webb S Lee RF Detection of '*Candidatus *Liberibacter asiaticus' in *Diaphorina citr *and its importance in the management of citrus huanglongbing in Florida Phytopathology 2008 98 4 387 396 10.1094/PHYTO-98-4-0387 18944186

[B9] Hodges A Philippakos E Mulkey D Spreen T Muraro R Economic impact of Florida's citrus industry, 1999-2000 University of Florida IFAS Economic Information Report 01-2 2001 1 14

[B10] Chung KR Brlansky RH Citrus diseases exotic to Florida: huanglongbing (citrus greening) Plant Pathology Department, Florida Cooperative Extension Service, Institute of Food and Agricultural Sciences, University of Florida 2009 http://edis.ifas.ufl.edu PP-201

[B11] Duan Y Zhou L Hall DG Li W Doddapaneni H Lin H Liu L Vahling CM Gabriel DW Williams KP Complete genome sequence of citrus huanglongbing bacterium, '*Candidatus *Liberibacter asiaticus' Obtained Through Metagenomics Mol Plant Microbe In 2009 22 8 1011 1020 10.1094/MPMI-22-8-1011 19589076

[B12] Nelson AJ Elias KS Arévalo GE Darlington LC Bailey BA Genetic characterization by RAPD analysis of isolates of *Fusarium oxysporum *f. sp. *erythroxyli *associated with an emerging epidemic in Peru Phytopathology 1997 87 12 1220 1225 10.1094/PHYTO.1997.87.12.1220 18945021

[B13] Wickert E Machado MA Lemos EGM Evaluation of the genetic diversity of *Xylella fastidiosa *strains from citrus and coffee hosts by single-nucleotide polymorphism markers Phytopathology 2007 97 12 1543 1549 10.1094/PHYTO-97-12-1543 18943714

[B14] Yuan X Morano L Bromley R Spring-Pearson S Stouthamer R Nunney L Multilocus sequence typing of *Xylella fastidiosa *causing Pierce's disease and oleander leaf scorch in the United States Phytopathology 2010 100 6 601 611 10.1094/PHYTO-100-6-0601 20465416

[B15] Coletta-Filho HD Bittleston LS Almeida RPP Spatial genetic structure of a vector-borne generalist pathogen Appl Environ Microb 2011 77 8 2596 2601 10.1128/AEM.02172-10 PMC3126377 21317251

[B16] Byrnes EJ III Li W Lewit Y Ma H Voelz K Ren P Carter DA Chaturvedi V Bildfell RJ May RC Emergence and pathogenicity of highly virulent *Cryptococcus gatti *genotypes in the Northwest United States PLoS Pathog 2010 6 4 e1000850 10.1371/journal.ppat.1000850 20421942 PMC2858702

[B17] Tomimura K Miyata S Furuya N Kubota K Okuda M Subandiyah S Hung TH Su HJ Iwanami T Evaluation of genetic diversity among '*Candidatus *Liberibacter asiaticus' isolates collected in Southeast Asia Phytopathology 2009 99 9 1062 1069 10.1094/PHYTO-99-9-1062 19671008

[B18] Adkar-Purushothama CR Quaglino F Casati P Ramanayaka JG Bianco PA Genetic diversity among '*Candidatus *Liberibacter asiaticus' isolates based on single nucleotide polymorphisms in 16S rRNA and ribosomal protein genes Ann Microbiol 2009 59 4 681 688 10.1007/BF03179208

[B19] Liu R Zhang P Pu X Xing X Chen J Deng X Analysis of a prophage gene frequency revealed population variation of '*Candidatus *Liberibacter asiaticus' from two citrus-growing provinces in China Plant Dis 2011 95 431 435 10.1094/PDIS-04-10-0300 30743331

[B20] Katoh H Subandiyah S Tomimura K Okuda M Su HJ Iwanami T Differentiation of "*Candidatus *Liberibacter asiaticus" isolates by variable-number tandem-repeat analysis Appl Environ Microbiol 2011 77 5 1910 1917 10.1128/AEM.01571-10 21239554 PMC3067300

[B21] Chen J Deng X Sun X Jones D Irey M Civerolo E Guangdong and Florida populations of '*Candidatus *Liberibacter asiaticus' distinguished by a genomic locus with short tandem repeats Phytopathology 2010 100 6 567 572 10.1094/PHYTO-100-6-0567 20465412

[B22] Nei M Tajima F Tateno Y Accuracy of estimated phylogenetic trees from molecular data. II. Gene frequency data J Mol Evol 1983 19 2 153 170 10.1007/BF02300753 6571220

[B23] Evanno G Regnaut S Goudet J Detecting the number of clusters of individuals using the software STRUCTURE: a simulation study Mol Ecol 2005 14 8 2611 2620 10.1111/j.1365-294X.2005.02553.x 15969739

[B24] Williams DA Overholt WA Cuda JP Hughes CR Chloroplast and microsatellite DNA diversities reveal the introduction history of Brazilian peppertree (*Schinus terebinthifoliu*) in Florida Mol Ecol 2005 14 12 3643 3656 10.1111/j.1365-294X.2005.02666.x 16202086

[B25] da Graça JV Biology, history and world status of huanglongbing Memorias del Taller Internacional sobre el Huanglongbing y el Psílido asiático de los cítricos 2008 Hermosillo, Sonora. México

[B26] Zhao XY Citrus yellow shoot (huanglongbing) in China: A review Proceedings International Society of Citriculture 1981 1 466 469

[B27] Beattie GAC Mabberley DJ Holford P Broadbent P de Barro P Huanglongbing: its possible origins, collaborative research in Southeast Asia, and developing incursion management plans for Australia Proceedings of the 2nd International Citrus Canker & Huanglongbing Research Workshop 2005 52

[B28] Capoor SP Decline of citrus trees in India Bull Natl Inst Sci India 1963 24 48 64

[B29] Raychaudhuri SP Nariani TK Lele VC Citrus die-back problem in India Proceedings of the First International Citrus Symposium 1969 3 1433 1437

[B30] Halbert SE The discovery of huanglongbing in Florida Proceedings of the 2nd International Citrus Canker and Huanglongbing Research Workshop 2005 Orlando Florida, USA 50

[B31] Bové JM Garnier M Citrus greening and psylla vectors of the disease in the Arabian peninsula Proceedings of the 9th Conference of the International Organization of Citrus Virologist 1984 109 114

[B32] Weinert MP Jackson SC Grimshaw JF Bellis GA Stephens PM Gunea T Kame MF Davis RI Detection of huanglongbing (citrus greening disease) in Timor-Leste (East Timor) and in Papua New Guinea Aust Plant Pathol 2004 33 135 136 10.1071/AP03089

[B33] Halbert SE Niblett CL Manjunath KL Lee RF Brown LG Establishment of two new vectors of citrus pathogens in Florida Proceedings of the International Society of Citriculture 9th Congress 2002 Alexandria, Virgina, USA: ASHS Press 1016 1017

[B34] Brlansky RH Dewdney MM Rogers ME 2011 Florida citrus pest management guide: huanglongbing (citrus greening) Plant Pathology Department, Florida Cooperative Extension Service, Institute of Food and Agricultural Sciences, University of Florida 2010 SP-43 http://edis.ifas.ufl.edu

[B35] Spann TM Atwood RA Dewdney MM Ebel RC Ehsani R England G Futch SH Gaver T Hurner T Oswalt C IFAS guidance for huanglongbing (greening) management Horticultural Sciences Department, Florida Cooperative Extension Service, Institute of Food and Agricultural Sciences, University of Florida 2010 HS1165 http://edis.ifas.ufl.edu

[B36] Benson G Tandem repeats finder: a program to analyze DNA sequences Nucl Acids Res 1999 27 2 573 58 10.1093/nar/27.2.573 9862982 PMC148217

[B37] Peakall R Smouse P GENALEX 6: Genetic analysis in Excel. Population genetic software for teaching and research Mol Ecol Notes 2006 6 288 295 10.1111/j.1471-8286.2005.01155.x PMC3463245 22820204

[B38] Raymond M Rousset F GENEPOP (version 1.2): population genetics software for exact tests and ecumenicism J Hered 1995 86 248 249

[B39] Tamura K Dudley J Nei M Kumar S MEGA4: Molecular evolutionary genetics analysis (MEGA) software Version 4.0 Mol Biol Evol 2007 24 8 1596 1599 10.1093/molbev/msm092 17488738

[B40] Pritchard J Stephens M Donnelly P Inference of population structure using multilocus genotype data Genetics 2000 155 945 959 10835412 10.1093/genetics/155.2.945 PMC1461096

[B41] Jakobsson M Rosenberg NA CLUMPP: a cluster matching and permutation program for dealing with label switching and multimodality in analysis of population structure Bioinformatics 2007 23 1801 1806 10.1093/bioinformatics/btm233 17485429

[B42] Rosenberg NA DISTRUCT: a program for the graphical display of population structure Mol Ecol Notes (2004) 2004 4 137 138

